# Supplementary material for: NanoHIVSeq: A Long-Read Bioinformatics Pipeline for High-Throughput Processing of HIV Env Sequences
Source: bioRxiv. 2026 Feb 19:2026.02.17.706429. Preprint. [Version 1] doi: 10.64898/2026.02.17.706429 (PMC12934658; doi:10.64898/2026.02.17.706429)
Supplement: Supplement 1 [file media-1.pdf]

A

HIV Strain	Accession Number
246_F3_C10_2	HM215279
398_F1_F6_20	HM215312
6535.3	AY835438
BJOX002000.03.2	HM215364
CAAN5342A2	AY835452
CAP45.2.00.G3	DQ435682
CE1176_A3	FJ444437
CE703010217_B6	FJ443575
CH119.10	EF117261
CNE55	HM215418
CNE8	HM215427
Du123.6	DQ411850
Du156.12	DQ411852
Du172.17	DQ411853
HIV_25710-2.43	EF117271
PV0.4	AY835444
QH0692.42	AY835439
RE-104541.67	AY835449
RHPA4259.7	AY835447
SS1196.1	AY835442
THR04156.18	AY835448
TRJ04551.58	AY835450
TRO.11	AY835445
WIT04160.33	AY835451
X1632_S2_B10	FJ817370
ZM109F.PB4	AY424138
ZM135M.PLIO	AY424079
ZM197M.PB7	DQ388515
ZM214M.PL15	DQ388516
ZM233M.PB6	DQ388517
ZM249M.PL1	DQ388514
ZM53M.PB12*	AY423984*

B

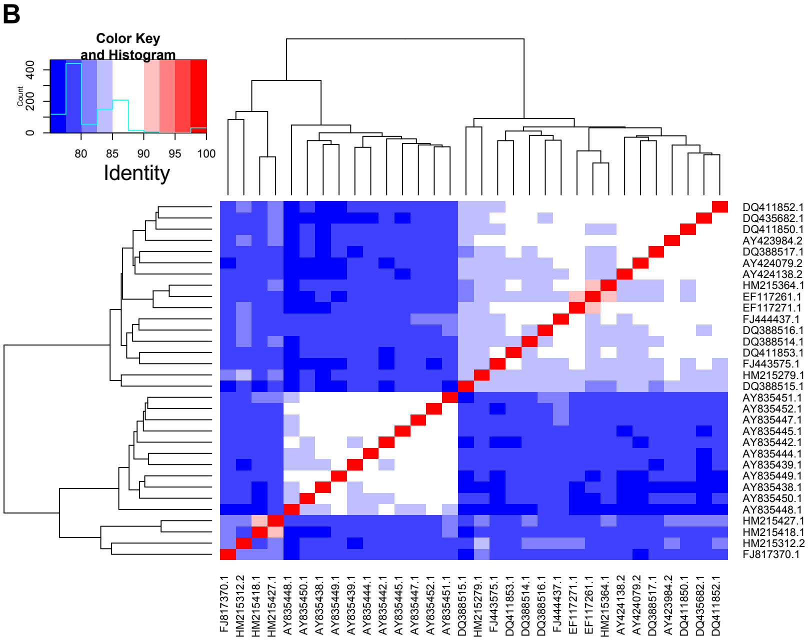

C

Read type	Calling model	Rawreads	Duplex_reads	Rawread_spikein	ENV_raw	ENV_unique
Simplex	FAST	634474	NA	13132	355564	355564
	HAC	506872	NA	17122	434374	433643
	SUP	526353	NA	18405	451143	293051
Duplex	HAC	711915	332353	5803	300857	233765
	SUP	748999	388631	6074	346102	181357

D

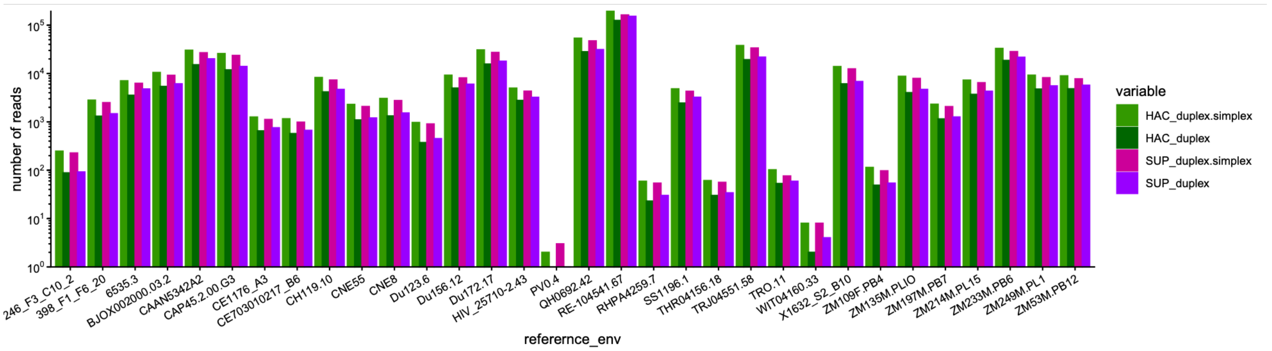

Figure.S1 Similarity of the 32 plasmids envs and ONT sequencing statistics.
(A) Accession numbers of the sequenced 32 envs. Note, ZM53M.PB12 has T1803G mutation in the plasmid compared to GenBank sequence. The mutation was confirmed by sanger sequencing.
(B) Heatmap to show sequence identity between the 32 plasmid envs.
(C) ENV sequencing run statistics using different basecalling model and read types.
(D) Raw read frequencies of the 32 references in four datasets. Blastn was used to assign each raw read to one of the 32 reference genes.

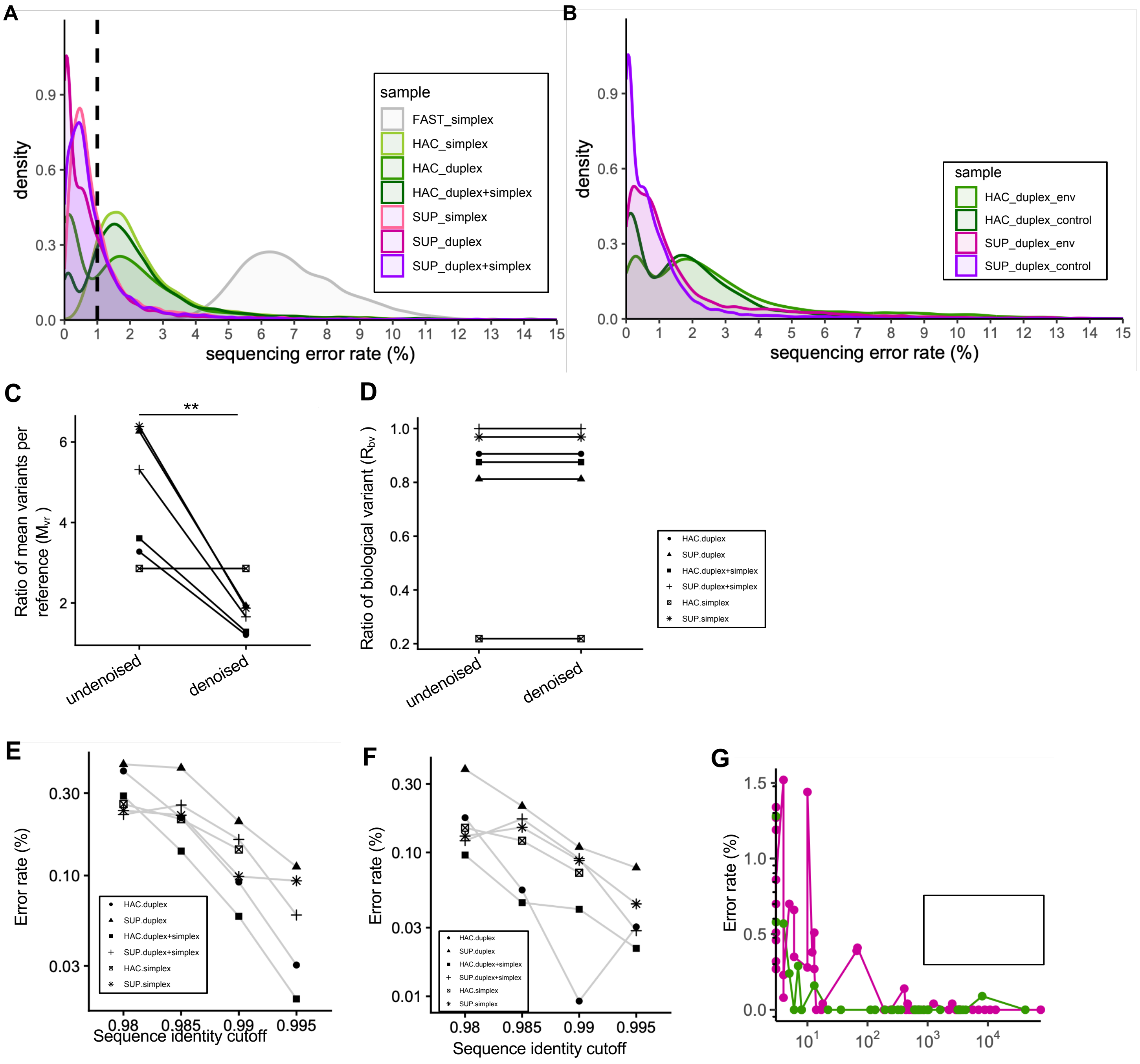

Figure.S2 Sequencing quality and env variant calling of 32 strains.

(A) Comparison of sequencing error rates of spikein lambda genome from duplex and simplex reads from fast, HAC, and SUP basecalling models. Reads with error rates higher than 15% were not shown.

(B) Comparison of sequencing errors of HIV-1 env and lambda genome. Reads with error rates higher than 15% were not shown.

(C) Denoise and chimera filter substantially reduced the ratio of mean variants per reference (M_{vr}). Usearch clustering with identity cutoff of 0.99 was used to generate consensus sequences. Paired T-test : p-value = 0.01.

(D) Denoise and chimera filter has no effect on the ratio of biological variants (R_{bv}). Usearch clustering with identity cutoff of 0.99 was used to generate consensus sequences.

(E) Error rates of curated envs from random subsampling of 100,000 duplex reads from HAC and SUP basecalled datasets. Error rates were calculated for curated envs with >=3, >=10, and >=15 read coverage. Ten subsampling repeats were generated.

(F) Error rates of curated envs from random subsampling of 150,000 duplex reads from HAC and SUP basecalled datasets. Error rates were calculated for curated envs with >=3, >=10, and >=15 read coverage. Ten subsampling repeats were generated.

(G) Sequencing error is reduced when sequencing depth increase. For both SUP and HAC reads, sequencing error is reduced substantially when sequencing depth increase to over 10 reads.

**, T-test p-value<0.01; ns, not significant.

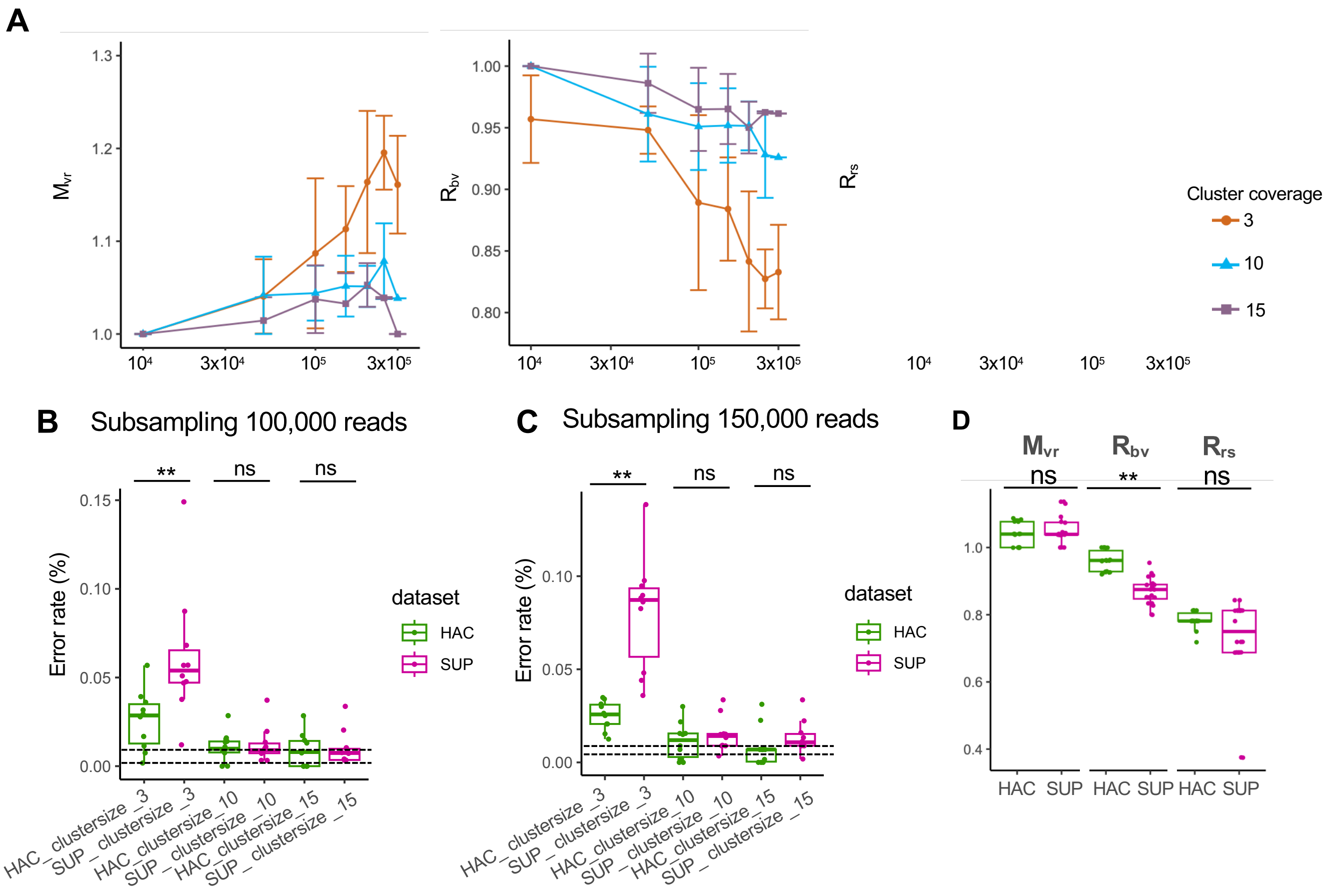

Figure.S3 Sequencing quality and env variant calling of 32 strains.

(A) Random subsampling revealed the distributions of R_{bv}, R_{rs}, and M_{vr} with ONT duplex reads increasing from 10,000 to 300,000.

(B) Error rates of curated envs from random subsampling of 100,000 duplex reads from HAC and SUP basecalled datasets. Error rates were calculated for curated envs with >=3, >=10, and >=15 read coverage. Ten subsampling repeats were generated.

(C) Error rates of curated envs from random subsampling of 150,000 duplex reads from HAC and SUP basecalled datasets. Error rates were calculated for curated envs with >=3, >=10, and >=15 read coverage. Ten subsampling repeats were generated.

(D) Comparison of R_{bv}, R_{rs}, and M_{vr} between HAC and SUP duplex datasets.

**, T-test p value<0.01; ns, not significant.

A

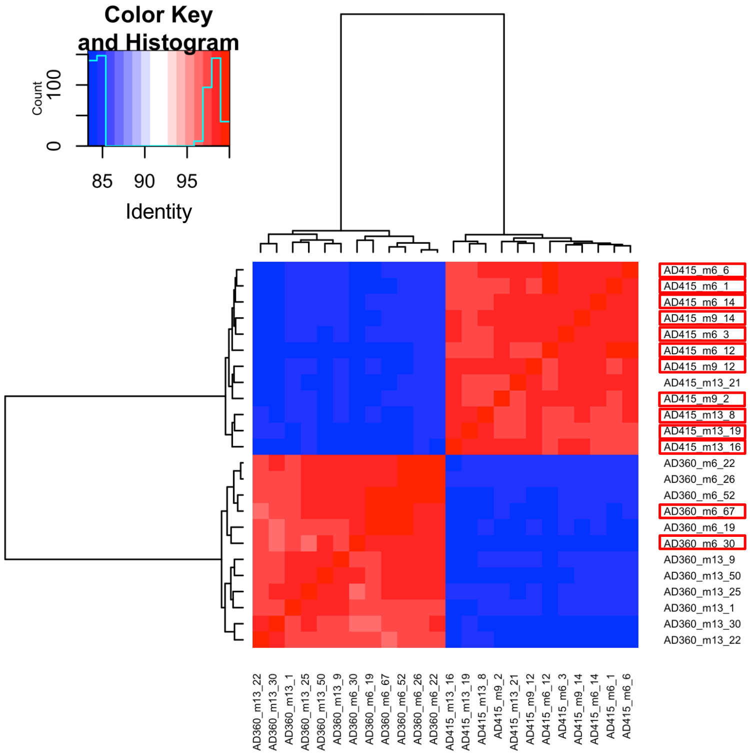

B

sampleID	Raw reads	ENV_raw	ENV_unique
repeate1	115189	49015	26477
repeate2	89846	38903	25963
repeate3	117919	17397	10752

C

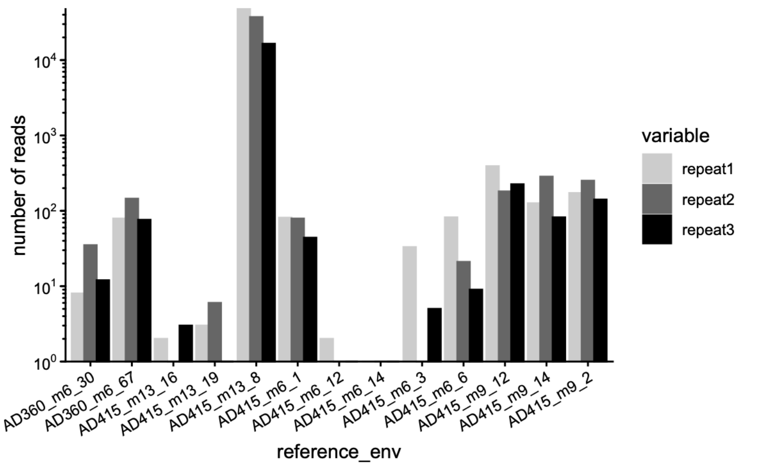

Figure.S4 Sequencing quality and env variant calling of 30 SGA.

(A) Sequence identity between env SGAs. SGAs detected in ONT datasets were highlighted in red.

(B) ENV sequencing run statistics. Unique reads are from usearch clustering with 0.99 identity. ONT reads are generated using duplex basecalling with the HAC model.

(C) Raw read frequencies of the 13 reference SGAs detected in three ONT repeats. Blastn was used to assign each raw reads to one of the 13 reference genes.

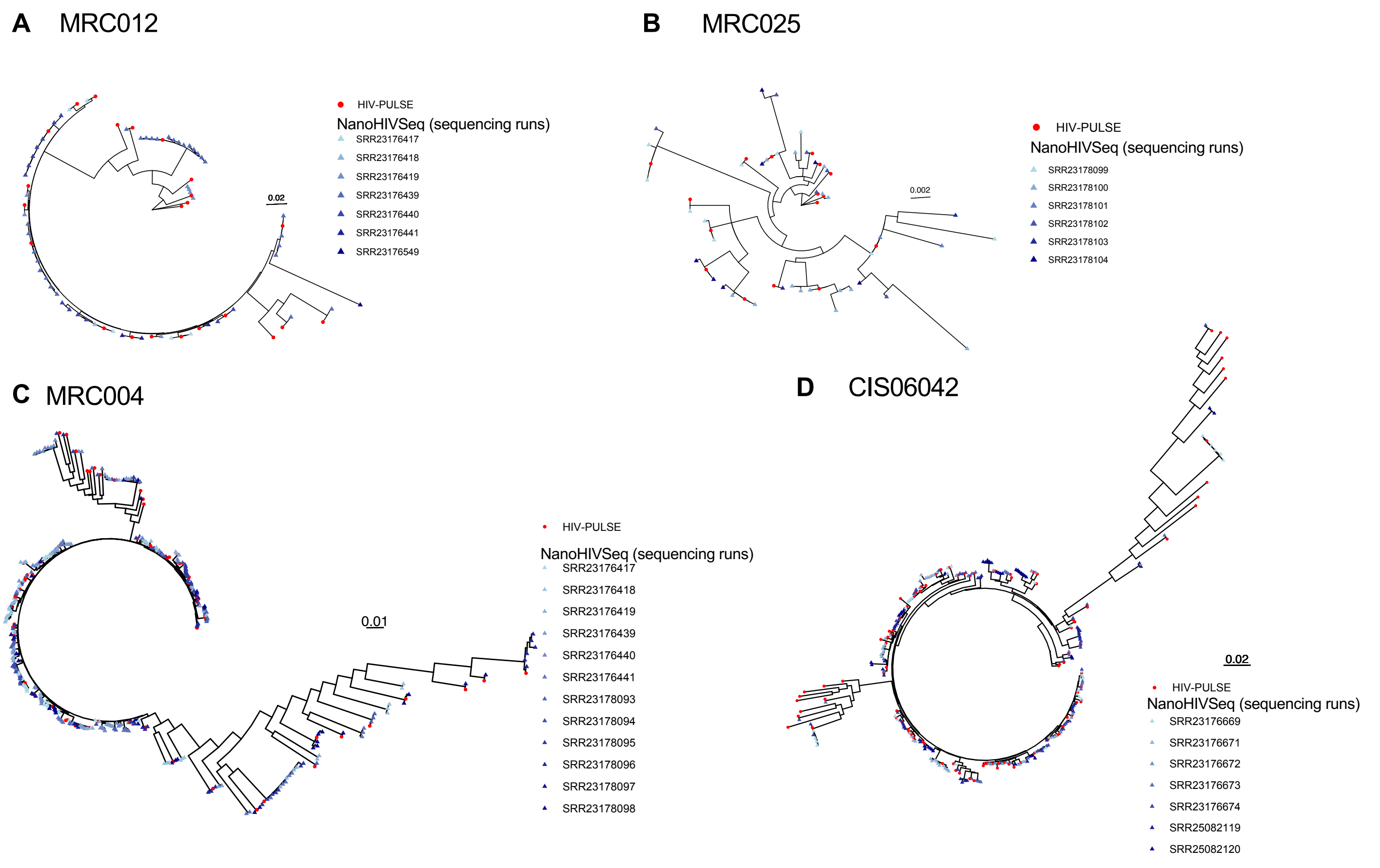

Figure.S5 Comparison of HIV-PULSE and NanoHIVSeq-derived unique env sequences.
(A) Donor MRC012 (B) Donor MRC025, (C) Donor MRC004, (D) Donor CIS06042. Each donor were sequenced multiple ONT runs whose SRA accession numbers are listed. NanoHIVSeq processed each ONT run separately. HIV-PULSE data are obtained from previous publication. The SRA run IDs were shown on the right.
